# microRNA Expression Profiling in Young Prostate Cancer Patients

**DOI:** 10.7150/jca.37842

**Published:** 2020-04-07

**Authors:** Vladimir A. Valera, Rafael Parra-Medina, Beatriz A. Walter, Peter Pinto, Maria J Merino

**Affiliations:** 1 Urologic Oncology Branch, National Cancer Institute, National Institutes of Health. Bethesda MD.; 2 Translational Surgical Pathology Section, Laboratory of Pathology, National Cancer Institute, National Institutes of Health, Bethesda MD.; 3 Faculty of Natural Science and Mathematics, Universidad del Rosario, Bogotá, Colombia

**Keywords:** MicroRNAs, prostate, early onset, young

## Abstract

MicroRNAs (miRNAs) are small, non-coding RNA molecules with multiple roles in many biological processes. Few studies have shown the molecular characteristics in younger prostate cancer (PCa) patients. In this study, we performed miRNA profiling in young PCa (EO-PCa) cases compared with PCa arising in older men (LO-PCa).

**Experimental Design**: Formalin-fixed, paraffin embedded tissue was used. miRNA was extracted for PCR array and NanoString methods. Relative miRNAs expression levels were obtained by comparing young vs older men, and young PCa tumor samples vs normal epithelium.

**Results**: miRNA profiling showed a different expression pattern in PCa arising in younger men, and young PCa tumoral and its normal counterpart. Nine miRNAs (hsa-miR-140-5p, hsa-miR-146a, hsa-miR-29b, hsa-miR-9, hsa-miR-124-3p, hsa-let-7f-5p, hsa-miR-184, hsa-miR-373, hsa-miR-146b-5p) showed differences in the expression compared to LO-PCa. Fourteen miRNAs were significantly up-regulated (miR-1973, miR-663a, miR-575, miR-93-5p, miR-630, miR-600, miR-494, miR-150-5p, miR-137, miR-25-3p, miR-375, miR-489, miR-888-5p, miR-142-3p), while 9 were found down-regulated (miR-21-5p, miR-363-3p, miR-205-5p, miR-548ai, miR-3195, 145-5p, miR-143-3p, miR-222-3p, miR-221-3p) comparing young PCa tumoral tissue compared to normal counterpart. The higher expression of miR-600 and miR-137 were associated with high Gleason score, extraprostatic extension and lymphatic invasion.

**Conclusion**: These results suggest that PCa in younger patients has a different expression profile compared to normal tissue and PCa arising in older man. Differentially expressed miRNAs provide insights of molecular mechanisms involve in this PCa subtype.

## Introduction

Prostate cancer (PCa) is the most common tumor in men and the fifth cause of cancer death. It is estimated that there were almost 1.3 million new cases of PCa and 359.000 associated deaths worldwide in 2018 1. The incidence of PCa has been increasing across the time. Between 1986 and 2008 the incidence of PCa in young men, defined as PCa arising in men under 55 years has been increased by 5.7-fold from 5.6 to 32 cases per 100.00 persons years (IC 95% CI 5.0-6.7), making PCa in young men an important emerging issue for public health 2,3. In 2012, 10% of men (241.740 persons) with newly diagnosed PCa were 55 years old or younger 4. Different risk factors as ethnicity, familiar history and genetic factors has been associated in this setting 5.

The genetic profile between young PCa and 'classical' or PCa arising in older men are different 3,6-9. Previous studies also have revealed different genetic alterations in young PCa, such as a greater number of single nucleotide polymorphism in the germinal DNA 6-8, different expressed genes involved in the inflammatory and immune-related pathways (CTL4, IDO1/TDO2) 9, and gene mutations in BRCA 1 and 2, and HOXB13 5. The relative risk to develop PCa in patients with BRCA 1 mutations has been reported as 1.8-4.5, while the relative risk reported in patients with mutations in BRCA 2 is 23, and patients with HOXB13 mutation have eightfold higher risk 5. PCa in young man with mutations in BRCA 1 and 2 has been associated with unfavorable prognosis, in contrast to patients with HOXB13 mutations were the genetic alteration has been associated with a favorable prognosis 5.

The molecular pathway of PCa in younger men is unknown. Weischenfeldt, et al. 7 recognized the important role of androgen regulated transmembrane serine protease 2 (TMPRSS2) gene fusion with the ERG gene. This fusion is a very early event in prostate oncogenesis and might be driven by increased androgen stimulation. Young PCa patients have a significantly increased tumor androgen receptor levels and positive correlation with ERG rearrangements. The frequency of ERG rearrangements in younger PCa patients is approximately 64% 10,11. TMPRSS2-ERG fusion positive cases are associated with loss of PTEN suppressor gene and TMPRSS2-ERG fusion negative cases with loss of 5q21 and 6q15 12.

Differences in clinical behavior between young and older PCa patients are controversial 3. Some studies report aggressive biological behavior and higher Gleason score in the young 3,11,13,14 while other studies report no significant difference in survival across age groups after prostatectomy, brachytherapy and radiation therapy 3,15,16. Schaefer et al. 10 observed that ERG-positive status was associated with low-serum PSA and lower prostate volume, while Huang et al. 11 further confirmed the ERG-positive status was associated with Gleason score and higher biochemical relapse rate but not with presurgical PSA levels, tumor volume, pathological stage, surgical margin or lymphovascular invasion.

MicroRNAs (miRNAs) are small, non-coding RNA molecules with multiple roles in many biological processes. They can prevent protein expression through cleavage of specific target mRNAs or through inhibition of their translation 17. Since the discovery of miRNAs, numerous studies have demonstrated their relevance in carcinogenesis of several cancers 18. Recently, it has been demonstrated the relevance of miRNAs in PCa as biomarkers in diagnosis, treatment, and prognosis.

In this study, we investigated whether PCa arising on younger men has a different microRNA profile compared to PCA in older men in order to further characterize its potential role in tumorigenesis, tumor progression and disease prognosis.

## Material and Methods

### Patient Samples

Formalin-fixed, paraffin embedded (FFPE) tissue samples from younger (age <55 y.o) patients with PCa and older (>55 y.o) PCa were retrieved from the surgical archives of the Laboratory of Pathology, National Cancer Institute, Bethesda, MD, USA after IRB approval. Samples without sufficient tumor tissue were excluded. Hematoxylin and eosin (H&E) stained slides were reviewed to confirm the diagnosis. Tumoral and normal tissue adjacent to the tumor (epithelial cells from the prostate glands) were selected from the same patients. The clinicopathological features were reviewed, including Gleason score, extraprostatic extension, margins, seminal vesicle, perineural invasion, lymphatic invasion, and pTNM (pathological tumor-node, metastasis) staging.

### microRNA Isolation

Isolation of total miRNA from FFPE specimens was performed as described previously 21,22. In brief, tumoral and normal tissue were manually microdissected under light microscope followed by miRNA isolation using the RecoverAll™ Total Nucleic Acid Isolations Kit (Ambion by Life Technologies, Foster City, CA, USA). The concentration of all RNA samples was quantified using NanoDrop 2000 (Thermo Scientific, USA). RNA concentration of samples used for profiling was normalized at 33 ng/µl following the recommendations from NanoString Technologies.

### microRNA expression profiling using PCR arrays

As we described previously 21, the extracted total RNA including miRNAs (10 ng/µl concentration) was first reverse transcribed into first strand cDNA using the RT2- miRNA First Strand Kit following manufacturer's recommendations (SA Biosciences, Rockville, MD). One µl cDNA per well was then mixed with SYBR Green qPCR Master Mix and placed into a 96-well PCR-array plate containing a panel of 88 mature miRNAs sequences. The arrays also contain appropriate small nucleolar RNA sequences that are used as housekeeping assays and quality controls. One µl was used in a 12 µl final volume reaction for Real-time PCR analysis on an Applied Biosystems Step-One Plus Real Time PCR system. Relative amounts were calculated by the ΔΔ CT method. Samples without good RNA quality were excluded in the statistical analysis.

### microRNA profiling using NanoString nCounter miRNA assay and data analysis

Total RNA samples were analyzed according to the manufacturer's instructions for the nCounter Human miRNA Expression Assay kit (NanoString Technologies, Seattle, WA). From each sample 100 ng from total RNA sample was used as input into the nCounter Human miRNA sample preparation. Hybridization was conducted for 16 h at 65°C. Subsequently, probes were purified and counted on the nCounter Prep Station. Each sample was scanned for 600 FOV (fields of view) on the nCounter Digital Analyzer. Data was extracted using the nCounter RCC Collector. The analysis was made as we described previously 22. miRNAs raw data was normalized for lane-to-lane variation with a dilution series of six spike-in positive controls. The sum of the six positive controls for a given lane was divided by the average sum across lanes to yield a normalization factor, which was then multiplied by the raw counts in each lane to give normalized values. For each sample, the mean plus 2 times the standard deviation of the 8 negative controls was subtracted from each miRNA count in that sample. Only miRNAs with non-negative counts across all samples were retained for downstream analysis. The relative miRNA levels were indicated as median fold changes (tumor/normal tissue) and a cutoff of 1.5-fold-change (up or down) was used.

### Assessment of prognostic significance of miRNAs associated with clinicopathological features

With PROGmir V2 we compared the overall survival, relapse-free survival, and metastasis-free survival of prostate adenocarcinoma patients with high and low expression of miRNAs associated with clinicopathological features. PROGmiR V2 is an online free tool that combines the prognostic data of miRNAs for different kinds of cancers 23.

### Statistical Analysis

Only mature miRNAs that showed at least a 1.5-fold change in expression are reported. p-values were calculated for each miRNA between the normal and tumor samples using the biological replicates and genes. They were considered differentially expressed and statistically significant if their p value was < 0.05. To compare relative miRNA fold changes between Young PCa and older PCa patients we used Mann-Whitney and Kruskal-Wallis non-parametric tests. According to edgeR, t-tests were carried out to compare the two groups (tumor vs. normal), p value was adjusted for multiple comparisons with the calculation of the false discovery rate (FDR) (<0.05). To evaluate differences between miRNA expression and clinicopathological features (Gleason score was categorized in low grade (3+3; 3+4) and high grade (4+3; 4+4;5+5)) we used t test. Supervised and non-supervised hierarchical clustering was conducted based on the Euclidean distance of miRNAs in samples using the Pheatmap package in R3.5.1. Analysis was performed using STATA SE 15 and R3.5.1.

## Results

### Characterization of studied population

In all, ten cases of young PCa and nineteen cases of older PCa patients were included. The clinicopathologic characteristics of the cases are shown in Table 1. The median age of young PCa was 46 years (range 40-55), 7 had low or intermediate risk Gleason score (3 + 3 or 3 + 4) and 3 had high-grade Gleason score (4 + 5 and 5 + 5). Perineural invasion was observed in seven patients, extraprostatic extension in three patients (high-grade Gleason score). While the average age of older PCa patients was 63 years (range 58-71), nine were low-grade Gleason score and ten were high-grade Gleason score. Perineural invasion was observed in 12 patients, extraprostatic extension and positive margins in six, and lymphatic invasion in four.

### microRNA expression profile comparing young PCa to older PCa patients

**Figure 1:**
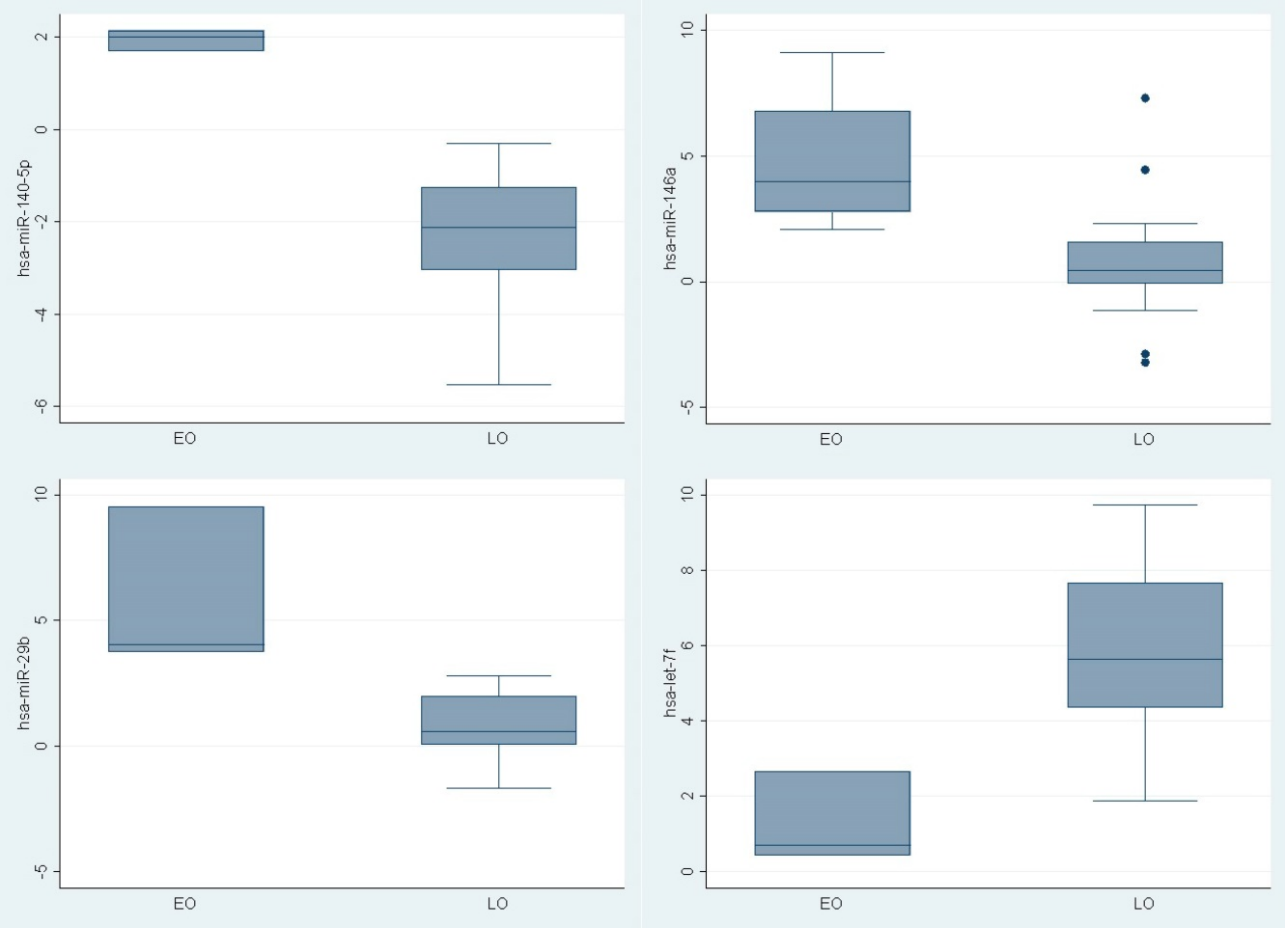
ΔΔ CT of miRNAs differentially expressed between young PCa and older PCa tumoral tissue

To determine if young PCa patients have a tumor specific pattern of miRNAs expression, the expression level of 88 mature miRNAs using PCR based assay was compared. Different expression was recognized in nine miRNAs between the groups (hsa-miR-140-5p, hsa-miR-146a, hsa-miR-29b, hsa-miR-9, hsa-miR-124-3p, hsa-let-7f-5p, hsa-miR-184, hsa-miR-373, hsa-miR-146b-5p) (Figure 1). Three were upregulated (Fold change >1.5) (hsa-miR-140-5p (p 0.008), hsa-miR-146a (p 0.01), hsa-miR-29b (p0.01)) in younger PCa patients and one (hsa-let-7f-5p ( p 0.02)) was downregulated (Fold change <1.5) (Table 2).

### microRNA expression profile between tumoral tissue to its normal counterpart in young PCa tumors

A panel of 800 miRNAs was analyzed in 6 young PCa patients. A t-test was performed to comparing the tumor versus its corresponding normal prostate epithelium. In total, 14 miRNAs were up-regulated ranging from 1.51-fold to 2.17 while nine miRNAs showed to be down-regulated ranging from -1.52-fold to -12.42-fold change. Only two miRNAs showed FDR <0.05 (hsa-miR-205 (FDR: 0.002) and hsa-miR-21-5p (FDR: 0.02) (Table 3). Among the 14 miRNAs up-regulated (hsa-miR-1973, hsa-miR-663a, hsa-miR-575, hsa-miR-93-5p, hsa-miR-630, hsa-miR-600, hsa-miR-494, hsa-miR-150-5p, hsa-miR-137, hsa-miR-25-3p, hsa-miR-375, hsa-miR-489, hsa-miR-888-5p, hsa-miR-142-3p), two miRNAs (hsa-miR-1973 and hsa-miR-93-5p) were the most prominently up-regulated (p<0.05).

Among the nine miRNAs down-regulated, three miRNAs (hsa-miR-21-5p, hsa-miR-363-3p, hsa-miR-205-5p) were the most prominently down-regulated (p<0.05).

**Figure 2:**
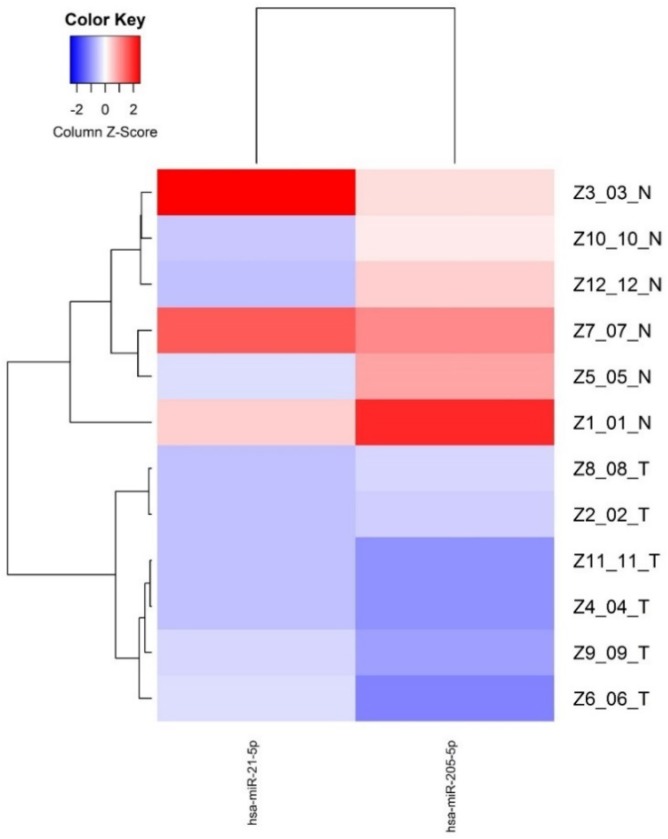
Differentially expressed miRNAs (FDR<0.05) between normal epithelium and tumor tissue from young PCa patients.

Supervised hierarchical clustering of the miRNAs down-regulated with FDR <0.05 was made based on miRNAs normalized expression, showing two groups separating normal and tumor epithelium mainly based the miR-205 expression, indicating a cancer specific expression pattern (Figure 2).

### microRNA expression and clinicopathological features

Using the expression profile data, we also evaluated the possible correlation between clinicopathological features and the expression of deregulated miRNAs in tumoral tissue. We analyzed the group of Young PCa patients for expression profiling regarding the Gleason score grade (low or high), extraprostatic extension, margins, seminal vesicle invasion, perineural invasion, lymphatic invasion, and pTNM stage. We found association between high levels of hsa-miR-575, hsa-miR-663a, hsa-miR-600, hsa-miR-137 with high grade Gleason score and presence of extraprostatic extension, as well the low levels of hsa-miR-143 (Table 4). In contrast, low levels of hsa-miR-221 was associated with low grade Gleason score and absence of extraprostatic extension. High levels of hsa-miR-137 and hsa-miR-600 were associated with presence of lymphatic invasion, while high levels hsa-miR-663, and low levels of hsa-miR-221 and hsa-miR-143 were associated with absence of lymphatic invasion. Low levels of hsa-miR-143 and high levels has-miR-1973 were associated with absence of perineural invasion (Table 4).

### Assessment of prognostic significance of miRNAs associated with clinicopathological features from PROGmir V2

**Figure 3:**
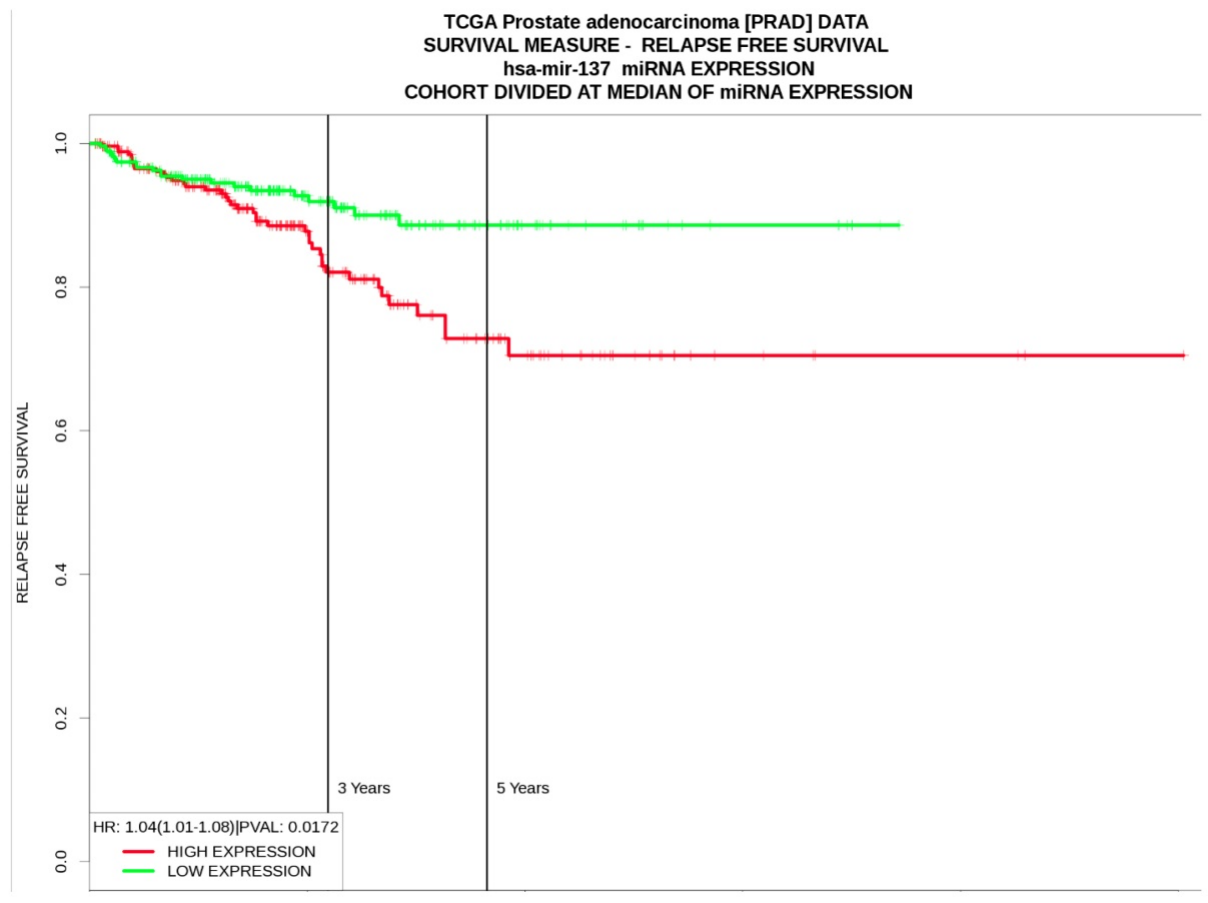
Relapse-free survival curves based on hsa-miR-137 expression levels in prostate cancer from PROGmiR V2.

According to the results from PROGmir V2, prostate cancer patients with high expression levels of hsa-miR-137 had significant poor relapse‐free survival (HR: 1.04 (1.01-1.08, p=0.01) (Figure 3). In contrast, high levels of hsa-miR-143 was associated as protective factor for relapse‐free survival (HR= 0.64 (0.48-0.84, p=0.001), same as hsa-miR-221 (HR= 0.68 (0.51-0.89, p=0.005) (Table 5).

## Discussion

In the present study, we observed a different expression profile of miRNAs in young PCa compared to older PCa patients (Table 2) and compared tumoral to normal tissue (Table 3), suggesting a cancer-specific miRNAs expression profile for Young PCa. Due to lack of studies about the miRNAs in young PCa, the current knowledge about its biology is limited. In the study by Diung et al 9 they found the different miRNAs expression between young PCa with GS 7 (3+4) and PCa in older patients. Like them, we observed differences in the expression of hsa-miR-146a, hsa-miR-9, hsa-miR-124-3p, hsa-miR-146b-5p. Other study tested the miRNA's expression in eleven young patients with PCa. They measured the expression of genomic alterations in younger PCa and found rearranged genes in androgen pathway. They focused on finding miRNAs that had as target to PTEN and identified 13 miRNAs (hsa-miR-17-5p, hsa-miR-19a-3p, hsa-miR-19b-3p, hsa-miR-20a-5p, hsa-miR-92a-3p, hsa-miR-106b-5p, hsa-miR-93-5p, hsa-miR-25-3p, hsa-miR-141-3p, hsa-miR-214-3p, hsa-miR-494, hsa-miR-222-3p, hsa-miR-21-5p) differentially expressed(> 1.5-fold change). In our study we also observed upregulation of hsa-miR-93-5p, hsa-miR-25-3p and hsa-miR-494, and downregulation of hsa-miR-222-3p when we compare tumoral vs normal tissue. Unlike of them, we found downregulation of hsa-miR-21-5p.

Among the miRNAs with differences between young and older PCa patients, we observed nine miRNAs with different levels of expression between them (hsa-miR-140-5p, hsa-miR-146a, hsa-miR-29b, hsa-miR-9, hsa-miR-124-3p, hsa-let-7f-5p, hsa-miR-184, hsa-miR-373, hsa-miR-146b-5p). Three were upregulated (Fold change >1.5) (hsa-miR-140-5p (p 0.008), hsa-miR-146a (p 0.01), hsa-miR-29b (p0.01)) in young PCa patients and one was downregulated (Fold change <1.5) (hsa-let-7f-5p ( p 0.02)). This in in agreement with published work that shows that hsa-miR-140-5p and hsa-let-7f-5p in PCa are upregulated 19,20,24 while hsa-miR-29b is downregulated 19,20. High expression levels of hsa-miR-140-5p has been observed mainly in metastatic PCa 24. hsa-miR-146a has shown to modulate androgen-Independent prostate cancer cells apoptosis through regulation of ROCK/Caspase 3 pathway 25. Furthermore, hsa-miR-29b has been observed downregulated in PCa 19,20 and may be involved in the epithelial-mesenchymal transition by the interaction with different targets such as e-cadherin, MMP-2, snail and twist 26.

Several miRNAs were observed dysregulated when we compared tumoral tissue versus tissue in Young PCa. Fourteen miRNAs were upregulated (Table 3). hsa-miR-93-5p has been reported overexpressed in PCa 27 and may are involved in cell proliferation, migration, invasion, block cell cycle, and promote the early apoptosis 27. In a previous study, it was found that hsa-miR-93 with hsa-miR-106b and miR-375 may downregulate CIC-CIC-CRABP1 and promote the progression of PCa 28. In young PCa, Weischenfeldt et al. found hypomethylation in the promotor region (R: -0.682, p<0.001) 7. On the other hand, hsa-miR-25-3p is part of a cluster with hsa-miR-93, hsa-miR-25 and hsa-miR-106b 29. This miR-106b-25 cluster promotes cell-cycle progression and hyperproliferation due to relationship with several actors in different oncological pathways such as PTEN, E2F1, and p21/WAF1 30-32. In PCa, the expression of this cluster is high and has been associated with tumor progression and metastasis 21,33. In a previous study, we had already observed upregulation of miR-25 in tumor cells versus normal epithelium 21. In young PCa the hypomethylation of the hsa-miR-25-3p promotor region (R: -0.625, p<0.001) has also been observed 7.

Among the miRNAs without previously reported association and whose biological functions have not been characterized in PCa are hsa-miR-1973, hsa-miR-575, hsa-miR-630 and hsa-miR-600. hsa-miR-575 and the hsa-miR-630 are proposed oncomiRNAs in other tumors such as gastric cancer, lung cancer, renal cell carcinoma, hepatocellular carcinoma, and breast cancer 34-40. hsa-miR-630 has different gene targets such as BCL-2, MTDH, YAP-1, SNAI2 37-39 involved in PCa oncogenesis 41-44. hsa-miR-575 has as a target BLID (BH3-like motif containing, BRCC2). BLID is a tumor-suppressor gene involved in DNA repair and gene integrity 40. In breast cancer, BLID inhibited cancer cell growth and metastasis via downregulating AKT pathway 40. In prostate cancer cell lines, it has been demonstrated that BLID induce a caspase-dependent mitochondrial pathway of cell death 45. Expression of hsa-miR-1973 has noted in other tumors as breast cancer, Hodgkin lymphoma and ovarian cancer 46-48. Other miRNA without previously reported in PCa is hsa-miR-600, this has been involved in breast cancer, lung and colorectal cancer 49-51.

Among the differentially expressed miRNAs, some were associated with Gleason score, extraprostatic extension and lymphatic invasion. high expression levels for hsa-miR-137 and hsa-miR-600 were associated with high Gleason score, presence of extraprostatic extension, and lymphatic invasion. hsa-miR-137 also was associated with poor relapse‐free survival (HR= 1.04). In the present study, we found that hsa-miR-137 is upregulated in young PCa, while in the literature has been reported downregulated and associated with recurrence following prostatectomy 52. hsa-miR-137 has a function as an androgen regulated suppressor of androgen signaling by modulating expression of an extended network of transcriptional coregulators 53.

## Conclusion

The present study supports the hypothesis that young PCa have a different miRNAs signature compared with normal tissue and older PCa patients. We present new miRNAs that may be involved in PCa pathogenesis (e.g. hsa -miR-1973, hsa-miR-575, hsa-miR-630, hsa-miR-3195), and present miRNAs with different expression (high or low) compared with previous studies (e.g hsa -miR-494, 150-5p, hsa -miR-137, hsa -miR-548ai, hsa -miR-21). In addition, we show that low expression of miR-600 and miR-137 were associated with clinicopathological features with poor prognosis and with poor relapse‐free survival. The identification of these miRNAs may provide insights into understanding this subtype of PCa and these miRNAs may prove to be useful as a biomarker in the diagnosis, prognostic as well in the development of new therapeutic approaches. More studies with larger sample size are needed to confirm the results presented in this study and to correlate them with clinicopathological outcomes in young patients with PCa.

## Figures and Tables

**Table 1:** Clinicopathological features of prostate cancer patients included in the study.

Variable	Young-PCa (N:10)	Older-PCa (N:19)	*p* value
Age, median (Years) (range)	46 (40-55)	63 (58-71)	
Gleason score groups			0.06
≤3+4	7	9	
≥4+4	3	10	
Extraprostatic extension			0.63
Yes	3	6	
No	7	13	
Positive margins			0.42
Yes	2	6	
No	8	13	
Seminal vesicle invasion			0.34
Yes	1	0	
No	9	19	
Perineural invasion			0.52
Yes	7	12	
No	3	7	
Lymphatic invasion			0.66
Yes	2	4	
No	8	15	
T Stage			0.24
pT2a ≤ pT2C	8	13	
pT3a≥ pT3	1	6	
N Stage			0.003
N0	8	4	
N1	2	15	
M Stage			1.000
M0	10	19	
M1	0	0	

**Table 2:** MicroRNAs differentially expressed in young PCa patients versus older PCa patients.

miRNA	Young PCa (mean, range)	Older-PCa (mean, range)	*p* Value
hsa-miR-140-5p	1.95 (1.69-2.15)	-2.28 (-5.53- -0.3)	0.008
hsa-miR-146a	4.78 (2.07-7.3)	0.79 (-3.21- 7.3)	0.01
hsa-miR-29b	5.77 (3.76-9.54)	0.8 (-1.66-2.8)	0.01
hsa-miR-9	1.21 (-3.38-4.11)	-4.84 (-9.63 - 1.9)	0.02
hsa-miR-124-3p	0.11 (-0.15-0.34)	2.55 (-7.03-8.61)	0.01
hsa-let-7f-5p	1.27 (0.43-2.67)	5.84 (1.88-9.73)	0.02
hsa-miR-184	0.09 (-1.14-1.27)	4.13 (-4.31-10.14)	0.02
hsa-miR-373	-1.43 (-4.48-0.41)	2.65 (-5.93-9.24)	0.03
hsa-miR-146b-5p	-0.69 (-1.32-0.03)	1.92 (-3.77-9.95)	0.04

**Table 3:** MicroRNAs differentially expressed in tumoral young PCa tissue versus normal epithelium.

miRNA	Fold change	*p* Value
hsa-miR-93-5p	1.87	**0.006**
hsa-miR-1973	2.17	**0.03**
hsa-miR-25-3p	1.6	0.07
hsa-miR-137	1.6	0.05
hsa-miR-575	2.01	0.09
hsa-miR-150-5p	1.61	0.09
hsa-miR-375	1.6	0.10
hsa-miR-663a	2.03	0.11
hsa-miR-142-3p	1.51	0.12
hsa-miR-630	1.83	0.16
hsa-miR-600	1.74	0.16
hsa-miR-888-5p	1.55	0.16
hsa-miR-489	1.6	0.17
hsa-miR-494	1.73	0.29
hsa-miR-205-5p	-4.81	**2.48E-06**
hsa-miR-21-5p	-12.42	**6.43E-05**
hsa-miR-363-3p	-5.47	**0.0089**
hsa-miR-145-5p	-1.61	0.05
hsa-miR-222-3p	-1.55	0.07
hsa-miR-3195	-1.87	0.08
hsa-miR-548ai	-2.83	0.09
hsa-miR-143-3p	-1.55	0.13
hsa-miR-221-3p	-1.52	0.12

**Table 4:** Correlation between clinicopathological features and miRNA expression levels

**Gleason score**			
	**High**	**Low**	**P-value**
hsa-miR-575	465.43	144.34	0.01
hsa-miR-663	91.95	26.56	0.04
hsa -miR-600	50.41	26.89	0.02
hsa -miR-137	88.25	68.24	0.03
hsa-miR-143	1356.32	532	0.01
hsa -miR-221	44.36	112.32	0.04
**Extraprostatic extension**			
	**Yes**	**No**	
hsa-miR-575	465.43	144.34	0.01
hsa-miR-663	91.95	26.56	0.04
hsa -miR-600	50.41	26.89	0.02
hsa -miR-137	88.25	68.24	0.03
hsa-miR-143	1356.32	532	0.01
hsa -miR-221	44.36	112.32	0.04
**Perineural invasion**			
	**Yes**	**No**	
hsa-miR-973	29.38	80.94	0.03
hsa -miR-143	824.02	1596.64	0.04
**Lymphatic invasion**			
	**Yes**	**No**	
hsa -miR-137	88.82	68.24	0.03
hsa -miR-600	50.41	26.89	0.02
hsa -miR-663	91.95	26.56	0.04
hsa -miR-221	44.36	112.32	0.04
hsa -miR-143	532.05	1356.32	0.01

**Table 5:** Prognostic significance of miRNAs with clinicopathological associations

miRNA's	Overall survival	Relapse-free survival	Metastasis-free survival
hsa -miR-137	HR: 0.98 (0.91-1.05)	HR: 1.04 (1.01-1.08)	HR: 1.05 (0.9-1.22)
hsa-miR-143	HR: 0.53 (0.27-1.06)	HR: 0.64 (0.48-0.84)	HR: 1.94(0.27-13.69)
hsa -miR-221	HR: 0.52 (0.27-1.02)	HR: 0.08 (0.51-0.89)	HR: 0.65 (0.19-2.3)
hsa-miR-663	HR: 0.96 (0.88-1.04)	HR: 0.97 (0.94-1.01)	HR: 0.91 (0.78-1.07)
